# High dose and low dose oxytocin regimens as determinants of successful labor induction: a multicenter comparative study

**DOI:** 10.1186/s12884-020-02938-4

**Published:** 2020-04-21

**Authors:** Melese Gezahegn Tesemma, Demisew Amenu Sori, Desta Hiko Gemeda

**Affiliations:** 1 Department of Obstetrics and Gynecology, Arsi University, Asella, Ethiopia; 2 Department of Obstetrics and Gynecology, Jimma University, Jimma, Ethiopia; 3 Department of Epidemiology, Jimma University, Jimma, Ethiopia

**Keywords:** Induction, High dose oxytocin, Low dose oxytocin, Successful induction, Failed induction, Induction to delivery time

## Abstract

**Background:**

Induction of labor by Oxytocin is a routine obstetric procedure. However, little is known regarding the optimal dose of oxytocin so as to bring successful induction. This study was aimed at comparing the effects of high dose versus low dose oxytocin regimens on success of labor induction.

**Methods:**

Hospital-based comparative cross-sectional study was conducted in four selected hospitals in Ethiopia prospectively from October 1, 2017 to May 30, 2018. A total of 216 pregnant women who undergo induction of labor at gestational age of 37 weeks and above were included. Data were entered into Epi-data version 3.1 and then exported to SPSS version 20 for cleaning and analysis. Chi-square test and logistic regression were done to look for determinants of successful induction. The result was presented using 95% confidence interval of crude and adjusted odds ratios. *P*-value < 0.05 was used to declare statistical significance.

**Result:**

The mean “Induction to delivery time” was 5.9 h and 6.3 h for participants who received high dose Oxytocin and low dose Oxytocin respectively. Higher successful induction (72.2% versus 61.1%) and lower Cesarean Section rate (27.8% vs. 38.9) were observed among participants who received low dose Oxytocin compared to high dose. Favourable bishop score [AOR 4.0 95% CI 1.9, 8.5], elective induction [AOR 0.2 95% CI 0.1, 0.4], performing artificial rupture of membrane [AOR 10.1 95% CI 3.2, 32.2], neonatal birth weight of <4Kg [AOR 4.3, 95% CI 1.6, 11.6] and being parous [AOR 2.1 95% CI 1.1, 4.0] were significantly associated with success of induction.

**Conclusions:**

In this study, Different oxytocin regimens didn’t show significant association with success of induction. But, high dose oxytocin regimen was significantly associated with slightly shorter induction to delivery time. Favourable bishop score, emergency induction, performing artificial rupture of membrane and delivery to non-macrosomic fetuses were positive determinants of successful induction. We recommend researchers to conduct multicenter research on a large number of patients that controls confounders to see the real effects of different oxytocin regimens on success of labor induction.

## Background

Induction of labor (IOL) refers to the iatrogenic stimulation of uterine contractions before the onset of spontaneous labor to accomplish vaginal delivery (VD) [1, 2]. Use of Oxytocin for labor induction is one of the most frequently used medications in obstetrics. This synthetic polypeptide hormone has been used to stimulate uterine contractions since 1950s after synthesized for the first time in 1953 by Vincent du Vigneaud [2, 3].

Oxytocin regimen can be classified as high-dose or low-dose based on different parameters. These are amount of starting dose, rate of incremental dose and intervals of escalation [4, 5]. The high-dose regimens varied across the different trials; starting doses ranged from 4 to 10 mili-unit/minute (mU/min), with increases in dose ranging from 4 to 7 mU/min and maximum rates ranging from 4 to 90 mU/min. The Low-dose regimen commences infusion at a range of 1–4 mU/min, with rate increases ranging from 1 to 2 mU/min and maximum rates ranging between 1 and 32 mU/min as mentioned in one systematic review [4]. Intervals of escalation of oxytocin doses vary from 15 to 60 min across the trials [1, 4–12].

Worldwide, there was neither agreement on standard oxytocin regimen nor strong evidence to recommend a particular dosage of oxytocin regimen for labor induction [5, 12]. However, one meta-analysis conducted in 1998 supports the use of a low-dose oxytocin infusion for IOL [3]. In Ethiopia, although very few studies were conducted to assess rate and determinants of induction success, there was no comparative study done to evaluate the effects of the two oxytocin regimens on success of induction. This study was, therefore aimed at comparing the effects of these oxytocin regimens on success of labor induction.

## Methods

### Study area, study period and study design

Comparative cross-sectional study was conducted in 4 hospitals namely Jimma University Medical Centre (JUMC), Shanan Gibe general hospital, Arbaminch General Hospital and Kuyu General Hospital prospectively from October 1, 2017 to May 30,2018. JUMC is the specialized teaching referral hospital where high dose oxytocin regimen was used for IOL while the three general hospitals used low dose oxytocin regimen as per national guideline.

### Study population

Pregnant women with singleton gestations who undergo induction of labor at gestational age (GA) of 37 weeks and above were recruited. Pregnant mothers with Intra Uterine Fetal Death (IUFD), critically ill pregnant mothers, pregnant mothers with lethal congenital anomaly, pregnancies complicated by cord prolapse, induced pregnancy for whom cesarean section (CS) was done for non-obstetric indication like social reason were excluded from the study.

### Sample size & sampling technique

The required sample size was determined by using double population proportion considering the following parameters: Proportion of CS among laboring mothers who received high dose of oxytocin (10.4%) and proportion of CS among laboring mothers who received low dose of oxytocin (25.7%) [7], 5% level of significance, power of 80% and 1:1 ratio of exposed to un-exposed. Considering 10% for non-response, 108 laboring mothers were recruited for each group. Thus, 108 pregnant women were recruited from JUMC while the rest 108 (36 from each) pregnant women were recruited from three general hospitals. All pregnant women who had undergone induction of labor during study period were recruited consecutively using inclusion criteria.

### Data collection, entry and analysis

Data were collected by trained midwifes using pretested structured questionnaire. Data like induction to delivery time, outcomes of induction (successful or failed), and mode of delivery, Oxytocin regimen, Bishop Scores and gestational age were collected from records of patient while socio - demographic data were collected by interviewing patients.

Data were edited and entered into Epi data version 3.1 and then exported to SPSS version 20 for cleaning and analysis. Chi-square test was conducted to compare participants who received high dose oxytocin and low dose oxytocin in terms of outcome data (route of delivery and outcomes of induction). Bivariate and multivariable logistic regression analyses were conducted to identify determinants of the successful induction. Findings were presented using 95%CIs of crude and adjusted odds ratios. *P*-value < 0.05 was used to declare statistical significance.

### Ethical considerations

Ethical clearance to conduct the research was obtained from institutional review board of Jimma University and written consent was obtained from study participants. All the information collected from the study participants were handled confidentially by omitting their personal identifiers and the data were used for the research purpose only. Participants were told by the language they understand that they have the right to participate in or withdraw from the study. In this research, the following operational definitions were used.

### Operational definitions

- **Successful Induction**: If a woman delivered vaginally with or without aid of instrument after induction with oxytocin.
- **Failed induction**: If a woman deliver by CS due to failure to acquire either adequate uterine contraction (≥3 contractions and duration lasting ≥40 s in 10 minutes period) or failed to show favorable cervical changes (reach at least 4 cm in dilatation and fully effaced) despite being on oxytocin drip for at least six to 8 hours.
- **Instrumental vaginal birth**: When vaginal delivery is effected by either vacuum or obstetric forceps.
- **Vaginal birth**: Is vaginal delivery without any assistance by instruments like vacuum or forceps
- **Induction to delivery time**: The time it takes the mother from starting of oxytocin to delivery of the fetus either vaginally or abdominally.
- **Low dose oxytocin regimen:** Initial dose of 2 mu/min increased by 2 mU/min every 30 min up to a maximum of 40 mU/minute.
- **High dose oxytocin regimen:** Initial dose of 6 mu/min increased by 6 mU/min every 20 min up to a maximum dose of 92 mu/min.
- **Favorable Bishop**: Those Bishop score having value of greater than six
- **Unfavorable Bishop**: Those Bishop score having value of less or equal to six [2]

## Result

### Socio-demographic, reproductive and obstetric characteristics of study participants

A total of 216 pregnant women were participated in the study in four hospitals. Mean age of study participants and mean gestational age at delivery was 26 years and 39.4 weeks respectively. These distributions were similar among the two study groups. One third 78 (36.1%) of the study participants were rural dwellers. Majority 176(82%) of the inductions were undergone on emergency basis. The top three indications for IOL in this study were premature rupture of membrane (PROM) 128 (59.3%), hypertensive disorders of pregnancy (HDP) 49 (22.7%) and post-term pregnancy 27 (12.5%) while the others accounted for 12 (5.6%). The indications for IOL were similar among the two study groups. Majorities of the participants enrolled in low dose group (LDG) 65(60.2%) had favorable Bishop score at initiation of induction unlike those enrolled in high dose group (HDG) which was observed only in 14(13%) (Table 1).

**Table 1:** Socio-demographic, Reproductive and Obstetric characteristics of the participants in four hospitals of Ethiopia

Variables		Type of oxytocin regimens		Total N**o** (%) ***N*** = 216 *n*(%)	***P***-Value
		High dose (*N* = 108) *n*(%)	Low dose (*N* = 108) *n*(%)		
**Age of respondent**	< = 19	4 (3.7)	5 (4.6)	9 (4.2)	.609
	20–29	76 (70.4)	81 (75.0)	157 (72.7)	
	> 30	28 (25.9)	22 (20.4)	50 (33.1)	
**Occupation of respondent**	House wife	66 (61.1)	57 (52.8)	123 (57)	.039
	Government employee	30 (27.8)	25 (23.1)	55 (25.5)	
	Merchant	7 (6.5)	7 (6.5)	14 (6.5)	
	Farmer	1 (0.9)	9 (8.3)	10 (4.6)	
	Others	4 (3.8)	10 (9.3)	14 (6.4)	
**Parity**	Nullipara	56 (51.9)	32 (29.6)	88 (40.7)	< .001
	Parous	52 (48.1)	76 (70.4)	128 (59.3)	
**Type of induction**	Elective	22 (20.4)	18 (16.7)	40 (18.5)	.483
	Emergency	86 (79.6)	90 (83.3)	176 (81.5)	
**Bishop score before induction**	Unfavorable	94 (87)	43 (39.8)	137 (63.4)	<.001
	Favorable	14 (13)	65 (60.2)	79 (36.6)	
**Gestational Age category**	Term	95 (88)	94 (87)	189 (87.5)	.837
	Post term	13 (12)	14 (13)	27 (12.5)	
**History of previous successful induction**	YES	5 (4.6)	11 (10.2)	16 (7.4)	.119
	NO	103 (95.4)	97 (89.8)	200 (92.6)	
**Misoprostol use for Ripening**	YES	40 (37)	57 (52.8)	97 (44.9)	.02
	NO	68 (63)	51 (47.2)	119 (55.1)	
**ARM done**	YES	27 (25)	18 (16.7)	45 (20.8)	.132
	NO	81 (75)	90 (83.3)	171 (79.2)	

### Labor outcomes (induction to delivery time, mean oxytocin concentration, rate of CS, rate of instrumental delivery, induction success)

Mean induction to delivery time was 5.9 h for HDG and 6.3 h for LDG while mean oxytocin concentration the laboring mother receiving at delivery were 77.6 mu/min and 22 mu/min respectively. Induction was successful in 61.1 and 72.2% of study participants among HDG and LDG while it was failed in 17.6 and 15.7% of mothers in the two groups respectively. Rates of instrumental delivery were 16 (14.8%) and 5 (4.6%) while that of CS were 42 (38.8%) and 30 (27.8%) among HDG and LDG respectively. Indications for CS in decreasing order were failed induction 19 (45%), Non reassuring fetal heart rate patterns (NRFHRP) 19 (45%) and CPD 4 (10%) among HDG while they were failed induction 17(56.7%), CPD 7(23.3%) and NRFHRP 6(20%) among LDG. NRFHRP as an indication of instrumental delivery was observed in 37.5 and 20% of participants enrolled in HDG and LDG respectively. Inadequate uterine contraction as a reason for failed induction was observed in 10 and 30% of HDG and LDG respectively (Table 2). Both successful induction (*p*-value = 0.083) and cesarean delivery (*P*-value = 0.083) didn’t show significant relation with use of different oxytocin regimens. Only instrumental vaginal delivery (*P*-value = 0.012) and vaginal delivery (*P*-value = 0.002) had relation with use of different oxytocin regimens (Table 2**).** Occurrence of Puerperal Sepsis showed significant relation with use of different oxytocin regimens (*P*-value =0.029).

**Table 2:** Labor outcome of pregnant women undergoing IOL with high dose and low dose oxytocin regimen in four hospitals of Ethiopia

Variables	Categories	Type of oxytocin regimen		Total (***N*** = 216)	***P***-Value
		High dose N_H_ = 108	Low dose (N_L_ = 108)		
**Reason for Failed induction**	No cervical change	17 (89.5)	12 (70.6)	29 (80.6)	
	Inadequate Uterine contraction	2 (10.5)	5 (29.4)	7 (19.4)	
**Indication for CS**	For failed Induction	19 (17.6)	17 (15.7)	36 (16.6)	
	For NRFHRP	19 (17.6)	6 (5.6)	25 (11.6)	
	For CPD	4 (3.7)	7 (6.5)	11 (5.1)	
**Indication for Instrumental delivery**	For Shortening SSOL	7 (6.5)	4 (3.7)	11 (5.1)	
	For NRFHRP	7 (6.5)	1 (0.9)	8 (3.7)	
	For Prolonged SSOL	2 (1.8)	0 (0)	2 (0.9)	
**Mode of delivery**	**VD**	50 (46.3)	73 (67.6)	123 (56.9)	.002
	**CS**	42 (38.9)	30 (27.8)	72 (33.3)	
	**Instrumental delivery**	16 (14.8)	5 (4.6)	21 (9.7)	
**Weight of neonate in grams**	2500–3999	103 (95.4)	89 (82.4)	192 (88.9)	.002
	> 4000	5 (4.6)	19 (17.6)	24 (11.1)	
**Successful induction**	Yes	66 (61.1)	78 (72.2)	144 (66.7)	.083
	No	42 (38.9)	30 (27.8)	72 (33.3)	
**Vaginal delivery**	Yes	50 (46.3)	73 (67.6)		.002
	No	58 (53.7)	35 (32.4)		
**Instrumental vaginal Delivery**	Yes	16 (14.8)	5 (4.6)		.012
	No	92 (85.2)	103 (95.4)		
**C/S Delivery**	Yes	42 (38.9)	30 (27.8)		.083
	No	66 (61.1)	78 (72.2)		
Occurrence of Puerperal Sepsis	Yes	0 (0.0)	6 (5.6)		0.029*
	No	108 (100)	102 (94.4)		
Occurrence of PPH Or Uterine Atony	Yes	3 (2.8)	3 (2.8)		1.00*
	No	105 (97.2)	105 (97.2)		
Occurrence of Uterine Hyper Stimulation	Yes	4 (3.7)	0 (0)		0.122*
	No	104 (96.3)	108 (100)		
Occurrence of Uterine Rupture	Yes	1 (0.9)	0 (0)		1.00*
	No	107 (99.1)	108 (100)		

*Fisher’s Exact Test was used

### Factors affecting success of induction

On Bivariate logistic regression analysis age, residence and family income of the respondent, previous history of successful induction, cervical ripening with misoprostol, type of oxytocin regimen, gestational age at delivery and neonatal weight did not show any kind of association with successful induction of labour. However, previous parity [COR 2.1, 95%CI 1.2, 3.7], Bishop score at initiation of oxytocin [COR 3.4, 95%CI 1.7, 6.7], type of induction [COR 0.4, 95%CI 0.2, 0.7] and performing ARM [COR 3.3, 95%CI 1.4, 7.9] were associated with successful IOL at *P*-Value < 0.05. (Table 3).

**Table 3:** Multivariate Logistic Regression of factors associated with success of induction in four hospitals of Ethiopia

VARIABLES	RESPONSE	Successful Induction		COR(95%CI)	*P* –value	AOR (95%CI)	*P*-Value
		Yes	No				
Age of respondent (in years)	< = 19	4 (44.4)	5 (55.6)	1		1	
	20–29	103 (65.6)	54 (34.4)	2.4 (0.6, 9.2)	.209	1.9 (0.4,9.9)	.43
	> 30	37 (74)	13 (26)	3.6 (0.8,15.3)	.088	2.6 (0.5,15.2)	.28
Residence	Urban	87 (63)	51 (37)	0.6 (0.3, 1.2)	.134	0.6 (0.3,1.29)	.19
	Rural	57 (73.1)	21 (26.9)	1		1	
Oxytocin regimen	High dose	66 (61.1)	42 (38.9)	0.6 (0.3, 1.1)	.084	0.6 (0.3,1.53)	.29
	Low dose	78 (72.2)	30 (27.8)	1		1	
Previous successful induction	YES	14 (87.5)	2 (12.5)	3.8 (0.8, 17.1)	.085	1.6 (0.3,9.02)	.58
	NO	130 (65)	70 (35)	1		1	
Gestational age at delivery	Term	129 (68.3)	60 (31.7)	1.7 (0.8, 3.9)	.194	0.6 (0.1,2.9)	.48
	Post-term	15 (55.6)	12 (44.4)	1		1	
Ripening with misoprostol	YES	59 (60.8)	38 (39.2)	0.6 (0.4, 1.1)	.101	0.4 (0.1,1.1)	.06
	NO	85 (71.4)	34 (28.6)	1			
Previous Parity	Parous	94 (73.4)	34 (26.6)	2.1 (1.2, 3.7)	.012	2.1 (1.1,4.0)	.024
	Nulliparous	50 (56.8)	38 (43.2)	1		1	
Bishop score before induction	Favorable	65 (82.3)	14 (17.7)	3.4 (1.7, 6.7)	.000	4.1 (2.0, 8.8)	.00
	Unfavorable	79 (57.7)	58 (42.3)	1		1	
Type of induction	Elective	19 (47.5)	21 (52.5)	0.4 (0.2,0.7)	.005	0.2 (0.1,0.5)	.001
	Emergency	125 (71)	51 (29)	1		1	
ARM done	YES	38 (84.4)	7 (15.6)	3.3 (1.4, 7.9)	.006	7.8 (2.7, 22.6)	.00
	NO	106 (73.6)	65 (90.3)	1		1	
Neonatal weight in gm	< 4000	132 (68.8)	60 (31.2)	2.2 (0.9, 5.2)	.071	4.3 (1.6, 11.6)	.005
	> 4000	12 (50)	12 (50)	1		1	

On multivariable logistic regression analysis, having favourable bishop score at initiation of oxytocin [AOR 4.0, 95% CI 1.9, 8.5], elective type of induction [AOR 0.2,95%CI 0.1,0.4], performing ARM [AOR 10.1,95%CI 3.2, 32.2], neonatal weight of < 4000 g [AOR 4.3, 95%CI 1.6, 11.6] and being parous [AOR 2.1, 95%CI 1.1,4.0] were found to be statistically significant. (Table 3).

## Discussion

### Determinants of successful induction

Being parous, having favorable Bishops score at initiation of oxytocin and performing ARM were significantly associated with increased success of induction by 2 times, 4 times and 8 times compared to nulliparous, unfavorable Bishop score and not performing ARM respectively. These results were in line with other study reports from Ethiopia [13–16]. This is because it is a well-established science that being parous, favorable cervical status and elective amniotomy or ARM were good predictors of successful induction of labor. Performing ARM strengthens the cascade of uterine contractions thus hastens labor and increase successful vaginal delivery. It was found that nulliparity had increased risk of failed induction by 1.5–3 times in other studies as well [13, 14, 17, 18].

Similarly, delivering to normal birth weight neonate compared to macrosomic neonate has increased success by 4 times. This might be justified by the fact that macrosomia is associated with labor dystocia and cephalo-pelvic disproportion thus ending in cesarean delivery. Our finding however, was not consistent with different literatures of the similar settings in Ethiopia that showed no association between neonatal birth weight outcome and induction success [13, 14, 16].

However, induction on elective basis compared to induction on emergency basis has reduced the induction success by 80%. This doesn’t show association with failed induction in study conducted by Woubishet et al. [13]. We expect higher successful induction of labor with elective induction than emergency induction. Because with elective induction one can buy time to ripen cervix till it gets favorable before initiating oxytocin thus increasing the success rate. But the finding of our study was opposite to this logic. This might be explained by the fact that majority of study participants (82%) were induced on emergency basis. On other hand, of all remaining elective inductions, 68% were induced for post term pregnancy. Post term was associated with decreased induction success as seen in different literatures [14, 16].

### Labor outcomes

Success of induction was lower among HDG compared to LDG (61.1% vs. 72.2%) while rate of CS was higher among HDG compared to LDG (38.8% vs. 27.8%). These findings were consistent with one meta-analysis that showed higher CS rate among HDG [3] and one cohort study done at Inova Alexandria Hospital (28% vs. 27%) [5]. However, the finding of our study was in contrary to one Cochrane review (18.8 vs 19.8) [12], one double masked randomized oxytocin trial (11.3% vs. 15%) [9], and other two studies (9% vs. 12%) [19] & (10.4% vs. 25.8%) [7] that showed higher CS rate among LDG. Although CS for failed induction occurred less frequently with the high-dose regimen (45.2% vs. 56.7%), CS for NRFHRP was performed more frequently (45.2% vs. 20%) compared to LDG.

In this study higher successful induction and lower CS rate among LDG were observed compared to HDG. We can raise many possible explanations why these occurred unlike other studies. Firstly, 60% of participants in LDG had favorable Bishop Score compared to HDG (only 13%) predicting higher successful induction and lower CS rate. Secondly, high dose oxytocin had statistically significant relation with NRFHRP in this study and mere occurrence of NRFHRP necessitating CS during labor might have reduced the possible number of successful vaginal deliveries if labor has to be continued. The fact that the number of mothers undergoing CS for NRFHRP among HDG was higher by 2.3 times than among the LDG (45% vs. 20%) may explain higher CS & lower successful induction observed among HDG.

Thirdly, although not statistically significant in this study, higher utilization of misoprostol for cervical priming among LDG (52.3% vs. 37%), presence of higher proportion of mothers with previous history of successful induction (10.2% vs. 4.6%) and significantly lower proportion of nulliparous women in LDG (29.6% vs. 51.9%) compared to HDG might have contributed to higher successful induction rate among LDG in our study. Because misoprostol use was standard of management as it increases success of induction. Lastly, the fact that centers with low oxytocin regimen use oral misoprostol for cervical priming before oxytocin induction in contrary to high dose center which initiate direct oxytocin induction for prolonged PROM, and PROM being major indication of induction (60%), might have contributed to higher induction success rate and thus lower CS rate among LDG .

Rate of failed induction was nearly the same among HDG (17.6%) and LDG (15.7%).This similarity in rate among the two groups was also seen in one cohort study comparing the two oxytocin regimen (4.3% & 5.1%) [5] and in one other double masked randomized oxytocin trial (6.0% & 6.1%) [9]. However, rate of failed induction was generally higher in our study compared to those studies. This might be due to the fact that the studies were following different protocols in relation to total duration of hours waited to diagnose failed induction. In this study failure to acquire either adequate uterine contraction or failed to show favorable cervical changes despite being on oxytocin drip for a period of six to 8 hours was used to diagnose failed induction. But other centers in literatures used to give more time ranging from 12 to 24 h as latent phase can usually be prolonged but ended in vaginal delivery [1].

### Mean induction to delivery Time & Mean Oxytocin to vaginal delivery

Mean “Induction to delivery” time for study participants were 5.9 h and 6.3 h for participants of HDG and LDG respectively while mean time elapsed from initiation of oxytocin to vaginal Delivery were 5.1 h and 6 h among HDG and LDG respectively. Mothers receiving high dose oxytocin regimen had slightly shorter duration of labor. This finding was similar to many literatures although majority of them showed significant shortening of induction to delivery time (2–3 h) compared to our study [3, 5, 6, 9, 10, 19]. Similar effect was found when using oxytocin for augmentation [4]. This might be due to frequent escalation of oxytocin dose among HDG compared to LDG (every 20 min Vs 30 min) till getting adequate uterine contraction or favorable cervical outcome.

The limitation to this study was confounders like parity, Bishop score, cervical ripening agent & fetal weight were not controlled so as to look the real effect of oxytocin regimen on induction success. The nature of hospitals being of teaching and public type was also another limitation to this study.

## Conclusion

Oxytocin regimen didn’t show any significant association with success of induction. But, high dose oxytocin regimen was significantly associated with slightly shorter induction to delivery time and higher utilization of instrument for delivery. Favourable bishop score, emergent type of induction, performing ARM and delivery to neonate weighing < 4 kg were positive determinants of successful induction. Thus we recommend that ripening cervix when condition allows, estimating fetal weight to exclude big baby and performing ARM before and during induction of labor. In addition we recommend researchers to conduct multicenter research on a large number of patients that controls confounders to see the real effect of different oxytocin regimens on induction success.

## Abbreviations

AOR: Adjusted odds ratio
ARM: Artificial Rupture of Membranes
CI: Confidence Intervals
CPD: Cephalo Pelvic Disproportion
COR: Crude odds ratio
CS: Caesarean Section
DPM: Drop Per Minute
HDG: High dose group
IOL: Induction of labor
JUMC: Jimma University Medical Centre
LDG: Low dose group
mu/min: Mili-unit/minute
NRFHRP: Non reassuring fetal heart rate patterns
PPH: Postpartum hemorrhage
PROM: Premature rupture of membrane
SPSS: Statistical Package for Social Scientists
SSOL: Second stage of labor
